# DPACFuse: Dual-Branch Progressive Learning for Infrared and Visible Image Fusion with Complementary Self-Attention and Convolution

**DOI:** 10.3390/s23167205

**Published:** 2023-08-16

**Authors:** Huayi Zhu, Heshan Wu, Xiaolong Wang, Dongmei He, Zhenbing Liu, Xipeng Pan

**Affiliations:** 1 School of Computer and Information Security, Guilin University of Electronic Science and Technology, Guilin 541004, China; 2001001034@mails.guet.edu.cn (H.Z.); 2000500927@mails.guet.edu.cn (H.W.); wwwxiaolong@mails.guet.edu.cn (X.W.); elh_hdm@163.com (D.H.); 2 School of Artificial Intelligence, Guilin University of Electronic Science and Technology, Guilin 541004, China

**Keywords:** multi-head self-attention, convolutional neural network, image fusion, gradient convolution, cross-modality interaction

## Abstract

Infrared and visible image fusion aims to generate a single fused image that not only contains rich texture details and salient objects, but also facilitates downstream tasks. However, existing works mainly focus on learning different modality-specific or shared features, and ignore the importance of modeling cross-modality features. To address these challenges, we propose Dual-branch Progressive learning for infrared and visible image fusion with a complementary self-Attention and Convolution (DPACFuse) network. On the one hand, we propose Cross-Modality Feature Extraction (CMEF) to enhance information interaction and the extraction of common features across modalities. In addition, we introduce a high-frequency gradient convolution operation to extract fine-grained information and suppress high-frequency information loss. On the other hand, to alleviate the CNN issues of insufficient global information extraction and computation overheads of self-attention, we introduce the ACmix, which can fully extract local and global information in the source image with a smaller computational overhead than pure convolution or pure self-attention. Extensive experiments demonstrated that the fused images generated by DPACFuse not only contain rich texture information, but can also effectively highlight salient objects. Additionally, our method achieved approximately 3% improvement over the state-of-the-art methods in MI, Qabf, SF, and AG evaluation indicators. More importantly, our fused images enhanced object detection and semantic segmentation by approximately 10%, compared to using infrared and visible images separately.

## 1. Introduction

Infrared and visible image fusion (IVF) has gained significant attention and is widely used in various applications [1]. Specifically, the effective fusion of shared and specific features from different modalities enables the generation of high-quality fused images, which, in turn, benefits downstream tasks, such as object detection [2,3,4], medical image processing [5,6], semantic segmentation [7,8,9], and pedestrian detection [10,11]. Although IVF has received much attention in various applications, IVF remains challenging due to the significant differences in appearance between these two image types. In infrared images, thermal target structures can be effectively highlighted, but these images often exhibit low contrast and blur properties. On the other hand, visible images have rich color and texture information, but they are easily affected by factors such as illumination and weather. Therefore, the effective fusion of these two different modalities into high-quality images still faces many technical difficulties.

Recently, there has been a surge in the development of deep learning-based IVF algorithms [12,13,14,15,16]. These methods typically involve feature extraction from the source images, the fusion of the extracted features, and the reconstruction of the fused features to obtain the final fused image [17]. Networks with strong feature extraction capabilities can usually synthesize fused images with better quality.

The main feature extraction method is currently the CNN-based auto-encoder structure, which is mainly divided into a shared encoder structure [18,19,20] and a dual-branch private encoder structure [21,22,23]. However, although the above-mentioned auto-encoder structure has good feature extraction ability, there are also some shortcomings. Firstly, the structure, based on the shared encoder, cannot distinguish the unique information in each mode. Secondly, the structure of the dual-branch private encoder often ignores the common features between modes, such as background and some large-scale features. In addition, the context-free CNN structure can only extract local features in a small service domain, and it is difficult to extract global information to generate higher-quality fusion images [15]. Moreover, many IVF networks may cause high-frequency information loss in image features when performing forward propagation [24]. Therefore, to address the above issues in IVF, this paper proposes a progressive feature extractor Cross-Modality Feature Extraction (CMFE). By introducing the CMFE module, compared with the structure of the shared encoder, our model can effectively extract the shared features between different modalities, while also better distinguishing the unique features between different modalities, to better realize IVF. Compared with the dual-branch private encoder, our structure can enhance the information interaction between different modalities to ensure thorough feature extraction, and our structure can better integrate the unique and shared features of modalities.

Vision Transformer has received extensive attention on many vision tasks. Therefore, many scholars have adopted Transformer-based methods in the feature extraction stage [13,15,25,26]. However, many transformer-based models are often limited by computational resources, the size of the input image, and weak local perception, which limits the timeliness and applicability of the IVF. To synthesize the respective advantages of Transformer and CNN architectures, we introduce ACmix [27], which integrates the flexibility of self-attention and the lightness of convolution. Therefore, we propose a complementary fusion network based on convolution and a multi-head self-attention mechanism to solve the IVF problem, integrating the advantages of CNN in extracting local information and computing portability and the ability of self-attention in context awareness and long-distance modeling. Compared with CNN-based architectures, our architecture has more powerful feature extraction capabilities and can better extract deep features in modalities. Additionally, compared with some Transformer-based architectures, our architecture has better flexibility (e.g., no fixed image size, small computational cost, etc.) and adaptability.

To this end, we propose Dual-branch Progressive learning for IVF with complementary self-Attention and Convolution (DPACFuse), such that the network can take into account the unique features and shared features of modalities, and integrate the respective advantages of CNN and a multi-head self-attention mechanism to better realize the IVF. The main contributions of this work can be summarized as follows:

- We propose a dual-branch progressive image fusion framework, based on complementary self-attention and convolution, for the IVF, which can take into account both global and local information of the source image to achieve better feature fusion.
- We propose Cross-Modality Feature Extraction (CMFE) to enhance the information interaction between modalities, while suppressing the loss of high-frequency information.
- Extensive experimental results on the public image fusion datasets, MSRS, RoadScene, and TNO, demonstrate that our method is superior to the current general fusion framework. Meanwhile, we investigate the facilitation of our fused images for object detection and semantic segmentation.

The remainder of this article is organized as follows. In Section 2, we mainly introduce the deep learning-based IVF methods. In Section 3, we introduce and describe, in detail, our fusion framework and the loss function used. In Section 4, we conduct a large number of experiments to verify the effectiveness of DPACFuse and also to explore the effect of IVF on downstream task promotion. In Section 5, we provide some conclusive proof.

## 2. Related Work

In this section, we briefly introduce the existing deep learning-based methods, mainly including CNN-based, AE-based, GAN-based, and Transformer-based methods.

### 2.1. CNN-Based and AE-Based Fusion Methods

In recent years, many CNN-based and AE-based methods have been widely used in the field of image fusion. Among them, the dual-branch structure, based on CNN and AE, greatly improves the fusion performance. For example, Tang et al. [21] proposed a dual-branch private encoder structure, namely SeAFusion, which combined a semantic segmentation network to learn more information, and to achieve better fusion results. Meanwhile, SeAFusion is a pioneer in combining IVF with downstream tasks. Additionally, Tang et al. [28] proposed an illumination-driven IVF network to solve the fusion problem in different lighting scenes. However, the designs of the network structures of the above two methods are too simple to effectively deal with complex situations. Another typical work is that of Res2Fusion, in [29], which describes a network with a dual-branch shared encoder structure, and which introduces Res2net and densely connected structures into the encoder to obtain multi-scale information. In the fusion layer, the fusion layer of double nonlocal attention models is used to realize image fusion. This method fully considers the problem of multi-scale information extraction and global modeling of the model, but it is difficult to deploy in the actual environment due to its high complexity and high computational cost. In addition, the shared encoder structure is also widely used in image fusion. For example, a typical method is DenseFuse [18]. The core concept of this method is to construct a deep neural network with dense connections, comprising an encoder (consisting of convolutional layers and dense layers) and a decoder (used for fusion). However, DenseFuse uses hand-designed fusion rules, so its results are not robust. In order to address the limitation of fusion rules that are designed manually, Li et al. proposed RFN-Nest [19] and NestFuse [20], wherein the former mainly utilized a residual fusion network to solve the problem, while the latter adopted the idea of combining spatial and channel attention mechanisms to solve the problem. Moreover, the IFCNN in [22], the PMGI in [30], and the U2Fusion in [31] proposed unified end-to-end networks to realize different fusion tasks.

Although many fusion methods based on CNN and AE have achieved good results, they usually adopt relatively simple CNN structures and hand-designed fusion rules, which limit the global modeling ability of the model and the ability to extract detailed information. At the same time, many methods lack information interaction in the process of feature extraction, which makes it impossible to fully extract more complementary information. In contrast, our method adopts the idea of cross-modality interaction to achieve image fusion, which helps to eliminate the mismatch and noise between different modalities and brings advantages in terms of improving the robustness of the fused image.

### 2.2. GAN-Based Fusion Methods

The Generative Adversarial Network (GAN) can learn the distribution characteristics of data and generate samples that conform to a specific distribution, which is also widely used in IVF. FusionGAN [32] was the first algorithm to apply this method to realize IVF. However, since only a single discriminator is used in FusionGAN, it cannot balance the information from the different modalities, leading to the loss of a lot of texture information in the fused images. In order to overcome the shortcomings of a single discriminator, Ma et al. [33] proposed a dual discriminator structure, namely DDcGAN, to achieve information fusion with image fusion. Moreover, Rao et al. [17] proposed AT-GAN, which introduced an intensity attention module and a semantic transition module to remove redundant information in infrared and visible images, respectively. At the same time, the quality assessment module is used to achieve the information balance between different modalities. In addition, infrared images have been greatly developed in various object detection tasks, such as pedestrian detection [11,34] and infrared small target detection [35,36]. However, due to the imaging characteristics of infrared images, the application scenarios of these methods are very limited. Therefore, there are many works [37,38,39] that combine IVF with object detection to overcome the limitations of using only a single modality. For example, Liu et al. [12] designed a GAN-based object perception network, TarDAL, which generated high-quality fused images and excellent detectors by including the IVF network and the object detection network in a bilevel optimization formulation.

Although GAN-based models have been widely used in the field of image fusion, the GAN-based fusion method emphasizes that discriminator learning simulates the distribution of the original image data, which may lead to poor image quality. At the same time, finding a way to balance the information from the different modalities is still a problem that needs to be studied.

### 2.3. Transformer-Based Fusion Methods

The transformer [40] structure is based on a multi-head self-attention mechanism, designed for sequence modeling and transduction tasks, and is known for its focus on long-term dependencies in data. Transformer has seen great success not only in NLP, but also in various visual tasks [41,42,43]. Many models based on Transformer have also been highly developed in the field of image fusion. For instance, Wang et al. [15] proposed a pure transformer fusion network, called SwinFuse, which used the powerful feature representation capability of the self-attention mechanism to perform image fusion. However, it uses a hand-designed fusion strategy, which does not perform well enough in handling fine-grained information. Additionally, Zhao et al. [44] introduced the Dual-branch Transformer and the structure of DenseNet (DNDT), which could consider more complete image information. In addition, inspired by the work of Swin Transformer [45], Ma et al. [13] proposed Swin Fusion, a network architecture for multimodal fusion. In addition, Rao et al. [46] proposed TGFuse, which embedded the Transformer in a GAN-based fusion network to achieve IVF. Furthermore, Qu et al. [26] proposed TransMEF for multi-exposure image fusion, which combined CNN and Transformer to obtain powerful local modeling and global modeling capabilities. However, this method is less flexible and can only input images of fixed size.

Although many transformer-based models perform well in many fusion tasks, many methods still suffer from poor flexibility and poor ability to model trans-membrane states, such as DNDT [44], TransMEF [26], and CGTF [47]. Furthermore, many transformer-based models are computationally expensive, while our method combines the excellent computational efficiency of CNN and the excellent global modeling ability of self-attention to better realize image fusion.

## 3. Methodology

### 3.1. Network Architecture

**Figure 1:**
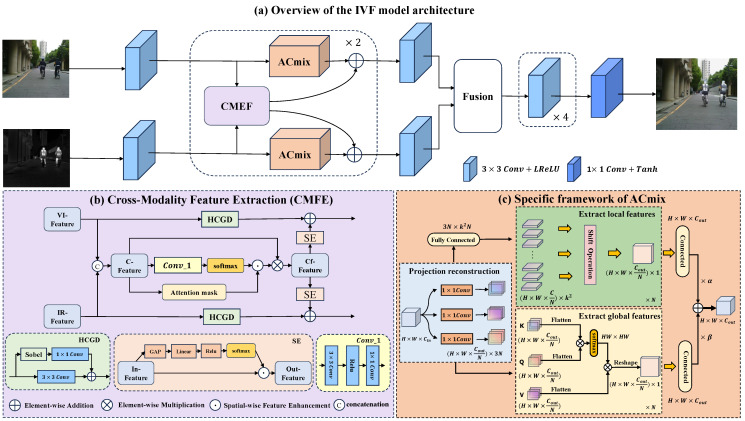
(**a**) Overview of the IVF model architecture. The feature extractor of the network consists of ACmix, CMEF, and 3×3 convolutional layers. Its feature reconstructor consists of 5 convolutional layers. (**b**) Cross-Modality Feature Extraction (CMFE). This mainly consists of a high-frequency convolution calculation based on Sobel operator (HCGD) and Squeeze-and-Excitation Network (SE). The HCGD adopts the idea of residual connection, and GAP and Linear in the SE schematic represent Global Average Pooling and Linear Function, respectively. (**c**) Specific framework of ACmix. This can be divided into three parts: Projection reconstruction, Extract local features, and Extract global features.

The network architecture of DPACFuse is illustrated in Figure 1a, and is composed of three main phases: feature extraction, feature fusion, and feature reconstruction. Given a pair of aligned infrared (IR) and visible (VI) images, denoted as $I_{ir}\in R^{H\times W\times C_{in}}$ and $I_{vi}\in R^{H\times W\times C_{in}}$, respectively, the fused image $I_{f}\in R^{H\times W\times C_{out}}$ is obtained through these phases.

In the feature extraction phase, we extract the specific features of the respective modalities separately using a dual-branch structure. First, we obtain shallow features $\left\{F_{ir}^{1},F_{vi}^{1}\right\}$ from the source image through a 3×3 convolutional layer. This can be expressed as:

(1) $$\begin{array}{c} \left\{F_{ir}^{1},F_{vi}^{1}\right\}=\left\{H_{se}\left(I_{ir}\right),H_{se}\left(I_{vi}\right)\right\} \end{array}$$

where $H_{se}\left(\cdot \right)$ represents a 3×3 convolutional layer, whose activation function is Leaky Relu and the stride is 1. The convolutions usually have stable optimization performance and are very good at early visual processing. At the same time, convolution has a strong local perception ability, which can effectively mine local information and map it to high-dimensional space.

Then, the ACmix is embedded in the respective branches of the IR and VI images to extract their respective specific features. At the same time, CMFE is deployed between the two modalities to extract their common features, thereby guiding the network to generate better images. We represent the feature extraction of the process in two stages. The intermediate features $\left\{F_{ir}^{2},F_{vi}^{2}\right\}$ obtained in the first stage can be expressed by the following formula:

(2) $$\begin{array}{cc} \left\{F_{ir}^{CF_{1}},F_{vi}^{CF_{1}}\right\} & =CMEF\left(F_{ir}^{1},F_{vi}^{1}\right)\\ \left\{F_{ir}^{AC_{1}},F_{vi}^{AC_{1}}\right\} & =\left\{ACmix\left(F_{ir}^{1}\right),ACmix\left(F_{vi}^{1}\right)\right\}\\ \left\{F_{ir}^{2},F_{vi}^{2}\right\} & =\left\{\left(F_{ir}^{CF_{1}}⊕F_{ir}^{AC_{1}}\right),\left(F_{vi}^{CF_{1}}⊕F_{vi}^{AC_{1}}\right)\right\} \end{array}$$

After obtaining the intermediate features $\left\{F_{ir}^{2},F_{vi}^{2}\right\}$ in the first stage, we use them as input for the second stage to obtain the output $\left\{F_{ir}^{3},F_{vi}^{3}\right\}$ in a similar manner to the first stage:

(3) $$\begin{array}{cc} \left\{F_{ir}^{CF_{2}},F_{vi}^{CF_{2}}\right\} & =CMEF\left(F_{ir}^{2},F_{vi}^{2}\right)\\ \left\{F_{ir}^{AC_{2}},F_{vi}^{AC_{2}}\right\} & =\left\{ACmix\left(F_{ir}^{2}\right),ACmix\left(F_{vi}^{2}\right)\right\}\\ \left\{F_{ir}^{3},F_{vi}^{3}\right\} & =\left\{\left(F_{ir}^{CF_{2}}⊕F_{ir}^{AC_{2}}\right),\left(F_{vi}^{CF_{2}}⊕F_{vi}^{AC_{2}}\right)\right\} \end{array}$$

Then, the features of these two modalities $\left\{F_{ir},F_{vi}\right\}$ are obtained by a convolutional layer $H_{de}\left(\cdot \right)$. The process can be expressed by the following formula:

(4) $$\left\{F_{ir},F_{vi}\right\}=\left\{H_{de}\left(F_{ir}^{3}\right),H_{de}\left(F_{vi}^{3}\right)\right\}$$

Finally, we reconstruct the fused image through the feature fusion and image reconstruction module. Since our feature extraction network has a strong enough extraction ability, we opted for a straightforward approach by employing the cascade fusion strategy to directly fuse the $F_{ir}$ and $F_{vi}$. The fusion process is represented as follows:

(5) $$F_{f}=H_{c}\left(F_{ir},F_{vi}\right)$$

where $F_{f}$ represents the fused feature, and $H_{c}\left(\cdot \right)$ represents the cascade on the channel dimension. Finally, we can obtain the output $I_{f}$ through the feature reconstructor $H_{R}\left(\cdot \right)$:

(6) $$I_{f}=H_{R}\left(F_{f}\right)$$

### 3.2. Specific Framework of ACmix

Due to the excellent context-aware ability of the multi-head self-attention mechanism and the lightness of convolution, we introduce ACmix. As shown in Figure 1c, it can be divided into: projection reconstruction, extract local features, and extract global features.

First, image features $I_{i}\in R^{H\times W\times C_{in}}$ are obtained and, after the projection reconstruction, local and global features are extracted, to obtain the output $F_{out}\in R^{H\times W\times C_{out}}$. In the projection reconstruction stage, the feature map $I_{i}$ is passed through three separate 1×1 convolutional layers, resulting in the generation of three feature maps $I_{i}^{1},I_{i}^{2},I_{i}^{3}\in R^{H\times W\times C_{out}}$.

In the extract local feature stage, the steps of this stage are different from the traditional standard convolution, that is, we first perform a linear projection of kernel weights, then translate according to the kernel position, and finally aggregate. Firstly, the three feature maps $I_{i}^{1},I_{i}^{2},I_{i}^{3}$ in the projection reconstruction stage, which are divided into *N* groups in the depth direction, and then reshaped to obtain a feature map with the dimensions of $R^{N\times \frac{C_{out}}{N}\times HW}$. After partitioning the feature maps into groups and reshaping them, the resulting feature maps are concatenated to create a new feature map $X\in R^{3N\times \frac{C_{out}}{N}\times HW}$. This concatenated feature map *X* is then fed through a lightweight fully connected layer to generate $Z\in R^{k^{2}N\times \frac{C_{out}}{N}\times HW}$. Then, *Z* is subjected to a reshaping operation and then a shift aggregation operation, which is realized by depthwise convolution. Specifically, *Z* is divided into *N* groups, and each group $Z_{l}\in R^{H\times W\times \frac{C_{out}}{N}}$ is used as a basic unit of convolution. Finally, the results of *N* groups are spliced to obtain the output $F_{conv}\in R^{H\times W\times C_{out}}$ of the convolution path. The entire extract local feature stage can be expressed as:

(7) $$\begin{array}{cc} X & =\mathrm{Connect}(R\left(I_{i}^{1}\right),R\left(I_{i}^{2}\right),R\left(I_{i}^{3}\right))\\ Z & =FC_{k}\left(X\right)\\ F_{conv} & =\overset{N}{\underset{l=1}{\|}}C_{dev}\left(Z_{l}\right) \end{array}$$

where Connect(·), R(·), and $FC_{k}\left(\cdot \right)$ denote the concatenation, reshape, and the light fully connected layer with a kernel size of *k*, respectively. The value $C_{dev}\left(\cdot \right)$ represents the convolution operation in depthwise convolution and $Z_{l}$ represents the input of group *l*th. The symbol || denotes the concatenation of the results obtained from all *N* groups, and the entire process corresponds to depthwise convolution with kernels of size 3. The processing of the extract local feature stage is the same as the traditional convolution operation.

In the extract global feature stage, the multi-head self-attention mechanism is adopted. Specifically, we divided the three feature maps $I_{i}^{1},I_{i}^{2},I_{i}^{3}$ obtained in the projection reconstruction stage into *N* groups (i.e., *N* attention mechanism heads) in the depth direction, and obtained the $Q\in R^{H\times W\times \frac{C_{out}}{N}}$, $K\in R^{H\times W\times \frac{Cout}{N}}$, $V\in R^{H\times W\times \frac{Cout}{N}}$ of each head. Then we flattened *Q*, *K* and *V* to obtain $Q^{\prime }\in R^{HW\times \frac{C_{out}}{N}}$, $K^{\prime }\in R^{HW\times \frac{C_{out}}{N}}$ and $V^{\prime }\in R^{HW\times \frac{C_{out}}{N}}$, and then used these as the inputs of the attention function:

(8) $$\begin{array}{c} \mathrm{Atention}\left(Q^{\prime },K^{\prime },V^{\prime }\right)=\mathrm{Softmax}\left(\frac{Q^{\prime }K^{\prime T}}{\sqrt{d_{k}}}\right)V^{\prime }\\ head_{j}=Atention\left(F\left(I_{i}W_{j}^{q}\right),F\left(I_{i}W_{j}^{k}\right),F\left(I_{i}W_{j}^{v}\right)\right)\\ F_{att}=\overset{N}{\underset{l=1}{\|}}R\left(head_{l}\right) \end{array}$$

where $W_{j}^{q}$, $W_{j}^{k}$, and $W_{j}^{v}\in R^{C_{in}\times \frac{C_{out}}{N}}$ are corresponding input projection weights (for ease of presentation, here, we include the process of the first stage). F(·) and R(·) denote the flatten and reshape operations, respectively. The symbols $d_{k}$, $head_{j}\in R^{H\times W\times \frac{C_{out}}{N}}$ and || denote the dimension of $K^{\prime }$, the output of the $j^{th}$ head, and the splicing of *N* heads, respectively. The symbol $F_{att}\in R^{H\times W\times C_{out}}$ represents the final output of the extract global feature stage, which is obtained by concatenating the outputs of the *N* self-attention heads.

Finally, the features extracted by the ACmix module can be expressed as the sum of the extract local feature path and the extract global feature path output, where the weights are determined by two learnable parameters α and β:

(9) $$\begin{array}{c} F_{out}=\alpha F_{conv}+\beta F_{att} \end{array}$$

### 3.3. Specific Framework of CMEF

To enhance the information interaction between modalities, as well as to suppress the loss of high-frequency information, we propose a CMEF module, the model of which is shown in Figure 1b. We introduce the module in two stages: feature combination and feature recombination.

In the feature combination stage, the input features $F_{1}\in R^{H\times W\times C_{in}}$, $F_{2}\in R^{H\times W\times C_{in}}$ are given. We first concatenate $F_{1}$ and $F_{2}$ to get the fusion feature $F_{cat}\in R^{H\times W\times 2C_{in}}$, and then obtain the common feature $F_{cf}\in R^{H\times W\times C_{out}}$ through the foreground-aware spatial attention and feature-level attention mask. The specific process is as follows:

(10) $$\begin{array}{c} F_{cat}=Concat\left(F_{1},F_{2}\right)\\ F_{cf}=\left[\left(\phi_{s}\left(F_{cat}\right)⊙\phi_{p}\left(Conv\_1(F_{cat})\right)\right)\right]F_{cat} \end{array}$$

where Concat(·) represents the operation of concatenating the features. The symbol $\phi_{s}\left(\cdot \right)$ stands for the foreground-aware spatial attention operation which is achieved by calculating the channel-wise maximum value of fusion features. The symbol $\phi_{p}\left(\cdot \right)$ represents the feature-level attention mask achieved by a Multiple Layer Perceptron (MLP), and Conv_1(·) followed by a 2-cls softmax operation. This feature-level attention mask means that $\phi_{p}\left(\cdot \right)$ can predict a re-scaling score to combine features from different modalities in such a way that the combined features are independent of the specific features.

In the feature recombination stage, it is well known that useless information has a huge impact on image fusion, which misleads the fusion direction of the model, resulting in distortion of the fused image. At this stage, we hope to obtain more common features and filter the interference of useless information as much as possible. Specifically, we integrate these shared features with the fine-grained information of their respective features through channel rescaling operations:

(11) $$\begin{array}{c} F_{out}^{ir}=SE\left(F_{cf}\right)⊕\phi_{GD}\left(\nabla F_{1}\right)⊕Conv\left(F_{1}\right)\\ F_{out}^{vi}=SE\left(F_{cf}\right)⊕\phi_{GD}\left(\nabla F_{2}\right)⊕Conv\left(F_{2}\right) \end{array}$$

where SE(·) represents the Squeeze-and-Excitation Network [48] (its framework is shown in Figure 1b), which can assign weights to each channel to effectively filter the impact of useless information on the fusion process. The symbol $\phi_{GD}\left(\cdot \right)$ refers to the gradient convolution operation, and ∇ stands for the gradient operator. Moreover, ⊕ and Conv(·) denote the operation of element-wise addition and 3×3 convolution, respectively. Finally, $F_{out}^{ir}$, $F_{out}^{vi}\in R^{H\times W\times C_{out}}$, respectively, add to their respective characteristics of the backbone network.

### 3.4. Loss Function

To minimize information loss and improve fusion performance, this paper employs three distinct loss functions for training the network: texture loss, intensity loss, and SSIM (Structural Similarity Index) loss. These loss functions constrain the network from different perspectives. The loss function used in our network can be represented as follows:

(12) $$L_{total}=\gamma_{0}L_{int}+\gamma_{1}L_{texture}+\gamma_{2}L_{ssim}$$

where $L_{int}$, $L_{texture}$, and $L_{ssim}$ represent the intensity loss, texture loss, and SSIM loss, respectively. The parameters $\gamma_{0}$, $\gamma_{1}$, and $\gamma_{2}$ are hyper-parameters to represent the contributions of the three losses to the entire loss, respectively.

The intensity loss emphasizes the preservation of pixel intensity information, and it helps the model better learn the overall brightness information and contrast characteristics. Intensity loss is defined as:

(13) $$L_{int}=\frac{1}{HW}{\left\|I_{f}-max\left(I_{ir},I_{vi}\right)\right\|}_{1}$$

where ${\left\|\cdot \right\|}_{1}$ denotes $l_{1}$ norm, and max(·) represents the maximum value in an element. By emphasizing the overall brightness and contrast characteristics, it enables the model to better understand and learn these important visual attributes.

The texture loss is a key component in image fusion, as it aims to preserve the intricate and fine-grained texture details during the fusion process. We define texture loss as:

(14) $$L_{texture}=\frac{1}{HW}{\left\||\nabla I_{f}|-max\left(|\nabla I_{ir}|,|\nabla I_{vi}|\right)\right\|}_{1}$$

where the symbol ∇ represents the Sobel gradient operator. The absolute value calculation, denoted by |·|, is applied to the gradient values to ensure that only positive magnitudes are considered. The value ${\left\|\cdot \right\|}_{1}$ represents $l_{1}$ norm, and max(·) selects the maximum value from the corresponding elements in the calculation.

The SSIM loss is employed to facilitate the learning of structural information by the model from the input images, and it also takes into account not only structure and contrast, but also illumination, which can be expressed as follows:

(15) $$L_{ssim}=\left(1-SSIM\left(I_{f},I_{ir}\right)\right)/2+\left(1-SSIM\left(I_{f},I_{vi}\right)\right)/2$$

## 4. Experiments

In this section, we provide specific details of the experimental implementation. We then compare DPACFuse with seven other methods. Finally, we demonstrate the outstanding performance of DPACFuse on downstream tasks.

### 4.1. Experimental Configurations

Datasets. The IVF experiments used three public datasets to verify our fusion method, which were MSRS [28], RoadScene [31] and TNO [49]. We trained our IVF network on the MSRS dataset, which contained 1083 pairs of registered images with semantic labels of nine typical scenes. In addition, we employed the MSRS test set (361 pairs), RoadScene (30 pairs), and TNO (30 pairs) as test datasets to comprehensively verify the performance of DPACFuse. Among them, the RoadScene dataset contained 221 image pairs that mainly focused on capturing typical traffic scenes, including roads, pedestrians, and vehicles. The TNO dataset consisted of multispectral night and day images depicting various military-related scenes.

Evaluation metrics and comparison methods. We used EN, SD, SF, MI, VIF, AG, Qabf, and FMI_pixel as evaluation metrics. In addition, higher metrics implied that the quality of the fusion image was better. Details on these evaluation metrics can be found in [50]. At the same time, we compared DPACFuse with the state-of-the-art methods, including DenseFuse [18], IFCNN [22], U2Fusion [31], SDNet [16], GANMcC [51], SwinFusion [13], and TarDAL [12].

Experimental setup. Our experiments were conducted on a computer equipped with one NVIDIA GeForce RTX 3090 GPU. The proposed method was implemented using the PyTorch platform. Moreover, all input images were normalized to [0, 1] before training. The following values were used for the hyperparameters of the experiment: the initial parameters α and β for the balanced convolution and self-attention paths were set to 1, respectively, the total number of self-attention heads was N=4, and the kernel size was k=3 for the fully connected layer. In addition, the hyperparameters were $\gamma_{0}=20$, $\gamma_{1}=20$ and $\gamma_{2}=1$ for balancing each loss function. The network parameters were updated using the Adam optimizer with a momentum term of (0.9, 0.999). The training was performed with a batch size of 2, an initial learning rate of 0.001, and a weight decay of 0.0002.

### 4.2. Comparative Experiment

#### 4.2.1. Qualitative Results

We selected two groups of images in the MSRS test set for subjective evaluation, wherein each group contained two typical scenes that were day and night.

**Figure 2:**
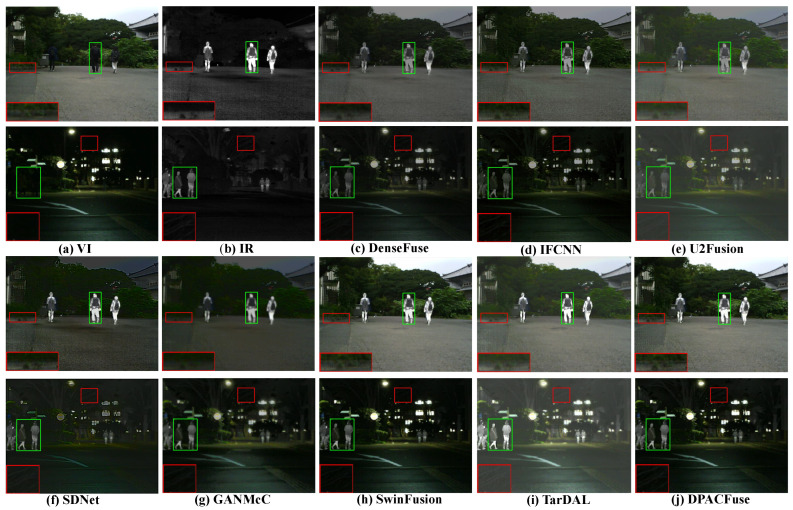
Qualitative analysis of DPACFuse with seven methods on 00634N (**top**) and 01356N (**bottom**) images from the MSRS dataset.

**Figure 3:**
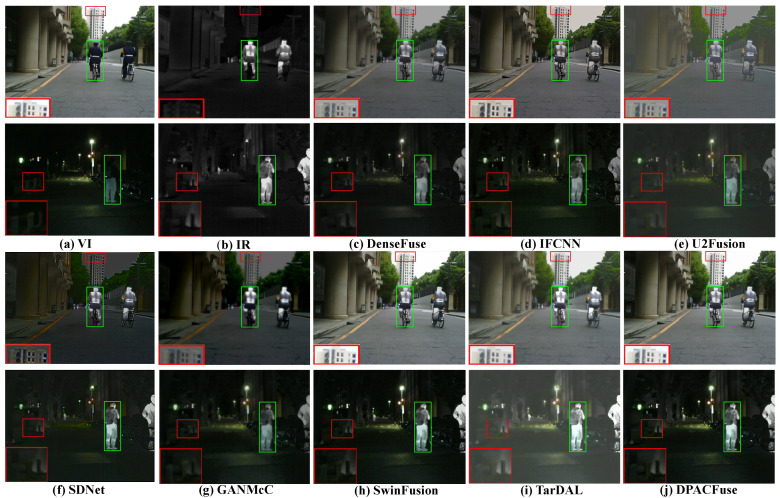
Qualitative analysis of DPACFuse with seven methods on 00537D (**top**) and 01012N (**bottom**) images from the MSRS dataset.

In the daytime scene with sufficient illumination, the VI image contained abundant texture detail and fully showed the environmental information. Although the ability of IR images to display the environment was limited, they could provide semantic information about the structure of thermal targets. By integrating this complementary information, the fusion image could provide comprehensive scene information, and could effectively enrich the semantic information. As presented in Figure 2 and Figure 3, due to the interference of useless information, the salient targets in DenseFuse, IFCNN, and U2Fusion methods weakened to varying degrees and could not maintain their original intensities. We highlighted salient regions with green boxes to illustrate this problem. Although SDNet and GANMcC could maintain the highlight intensity of infrared targets, their performances in retaining texture information was poor, and we illustrated the problem by zooming in on the areas with red boxes. In addition, compared with SwinFusion and TarDAL, DPACFuse not only retained more detailed information, but also better preserved the edge information, as can be seen from the enlarged floors, as well as the steps.

In the dark scene with insufficient illumination, due to the influence of illumination, VI images could only provide limited environmental information, and objects in them were not easy to identify, while IR images were not sensitive to illumination. Therefore, adaptive realization of the IVF in the case of the dark scene was very important, whilst being very challenging. As presented in Figure 2 and Figure 3, all methods could effectively construct the scene information, but there were great differences between different algorithms. Excepting our DPACFuse, SwinFusion, and TarDAL, the other methods failed to maintain the highlighting of thermal targets in infrared images, which we illustrated with the green boxes. In addition, DPACFuse was better than the other methods, such as SwinFusion, in maintaining details, which we illustrated by magnifying the red area.

Overall, the experimental results highlighted the superior performance of DPACFuse in both daytime and dark scenes. It effectively preserved texture details, edge information, and the saliency of thermal targets, demonstrating its superior ability to perform IVF in a variety of environmental conditions.

#### 4.2.2. Quantitative Result

**Figure 4:**
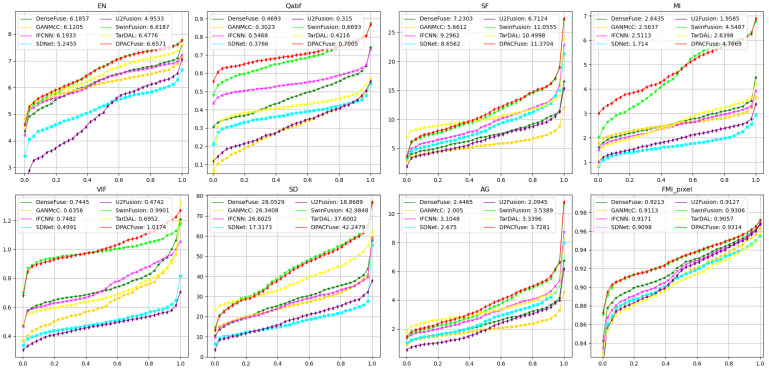
Quantitative comparison of eight methods on the MSRS test set. The *x*-axis represents cumulative distribution and the *y*-axis represents the values of the metric. The point (x,y) on the curve represents the measurement value of the x×100 percent of image pairs not exceeding the value of *y*. The average value is shown in the legend.

Figure 4 displays the quantitative results of the eight evaluation indicators on the MSRS test set. DPACFuse demonstrated superior performance in nearly all metrics, showcasing its ability to effectively extract information from the source images and its versatility across various complex scenes. The best EN, MI, and FMI_pixel indicated that our fused image contained the most information, and the highest Qabf and AG indicated that our fused image retained the most edge information. In addition, the highest SF and VIF illustrated the best visual effect was presented by our fused image. Although DPACFuse slightly lagged behind SwinFusion in terms of the SD metric, the difference was not significant, which meant that our fused images had good contrast.

### 4.3. Generalization Experiment

Generalization ability is also an important metric to evaluate a model. We trained the model on the MSRS dataset and verified the generalization ability of DPACFuse on the RoadScene and TNO datasets.

#### 4.3.1. Results of RoadScene

**Figure 5:**
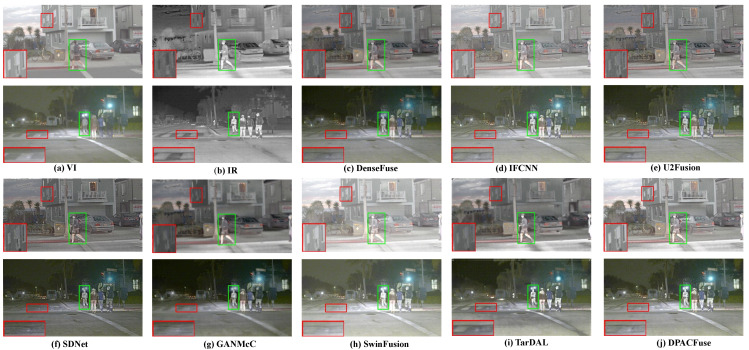
Qualitative analysis of DPACFuse with seven methods on FLIR_06307 (**top**) and FLIR_03952 (**bottom**) images from the RoadScene dataset.

Qualitative analysis. We selected two scenes, day and night, to assess the fusion results, and the visualized results are shown in Figure 5. Observing the results of the daytime scene, we can see that almost all the algorithms suffered from the interference of useless information, among which DenseFuse, U2Fusion, SDNet, GANMcC, and TarDAL were most affected, losing a lot of texture information. We illustrated this problem by zooming in on the red area. In addition, the intensity of the infrared targets of SDNet and GANMcC also weakened to varying degrees, while SwinFusion experienced a decrease in its overall contrast, due to the influence of illumination, which we illustrate by the green box. Except for our DPACFuse and SwinFusion, the other methods weakened in the overall pixel intensity and could not maintain the original pixel intensity.

In the dark scene, it can be seen that DenseFuse, U2Fusion, SDNet, GANMcC, and TarDAL lost a lot of texture details, such as the outline of background leaves and the zebra crossing on the ground. In addition, the salient targets of DenseFuse, SDNet, and GANMcC were severely disturbed by useless information and could not maintain the original pixel intensity. DPACFuse and SwinFusion were only disturbed by a small amount of useless information.

**Figure 6:**
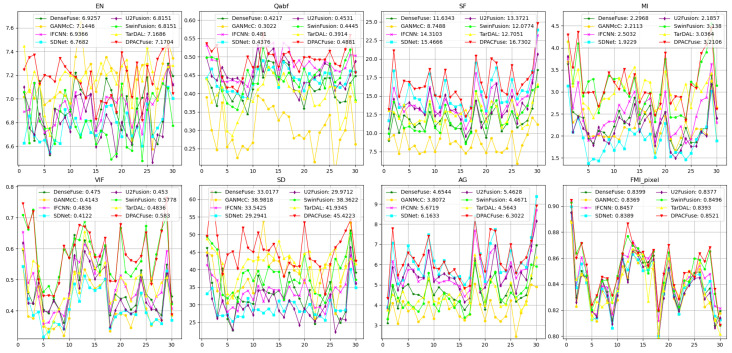
Quantitative comparison of eight methods on the RoadScene test set. The *x*-axis represents image pairs and the *y*-axis represents the values of the metric. The point (x,y) in the image represents the measurement *y* for the *x*th pair of images. The average value is shown in the legend.

Quantitative analysis. As shown in Figure 6, DPACFuse achieved the highest scores in all indicators, which meant that the fused image generated by DPACFuse not only maintained a lot of information and texture details, but also had the highest contrast and the best visual quality.

The excellent performance of DPACFuse on the RoadScene dataset fully demonstrated the adaptability of our method to various complex traffic scenes, and also proved that DPACFuse has good generalization ability.

#### 4.3.2. Results of TNO

**Figure 7:**
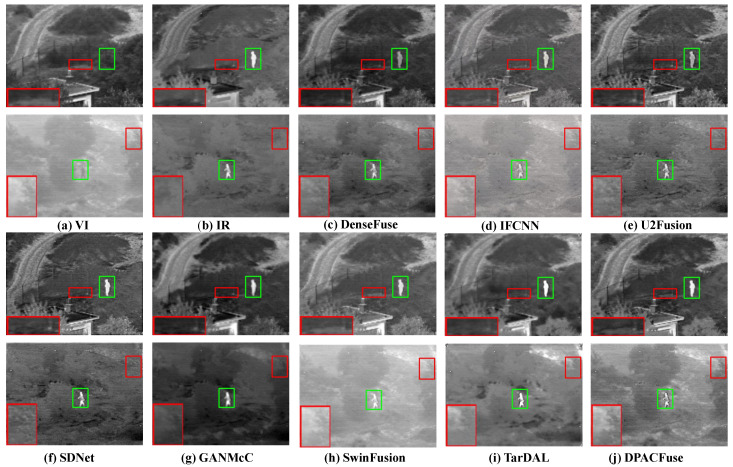
Qualitative analysis of DPACFuse with seven methods on two representative images from the TNO dataset.

Qualitative analysis. As depicted in the green boxes in Figure 7, DenseFuse, U2Fusion, and IFCNN weakened the strength of salient targets to different extents, with the first two being the most obvious. In addition, GANMcC blurred the contour of the salient targets. Excepting our method and SwinFusion, the fused images of the other methods were affected by other useless spectral information and could not effectively present the texture information, such as the bushes and fences in the red region. It is worth noting that, although SwinFusion had good fusion performance, the fused images suffered from whitening. On the whole, DPACFuse not only excelled in highlighting salient objects, but also effectively preserved the original texture information from the input images.

**Figure 8:**
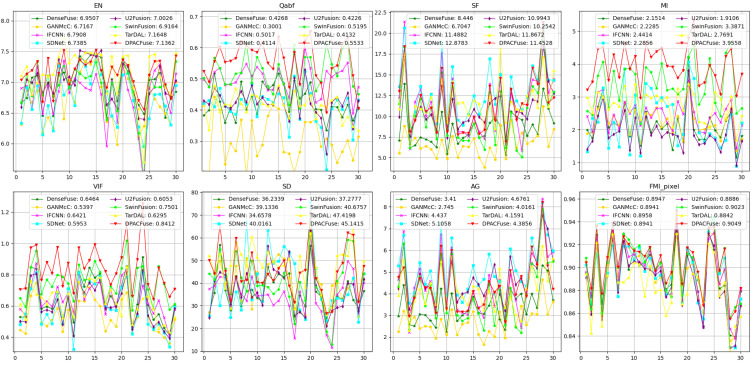
Quantitative comparison of eight methods on the TNO test set. The *x*-axis represents image pairs and the *y*-axis represents the values of the metric. The point (x,y) in the image represents the measurement *y* for the *x*th pair of images. The average value is shown in the legend.

Quantitative analysis. The results depicted in Figure 8 illustrate that DPACFuse achieved the highest scores in Qabf, MI, VIF, and FMI_pixel metrics. In addition, DPACFuse was also ahead of all the methods, excepting TarDAL, in two metrics: EN and SD. Taking the above analyses together, DPACFuse exhibited excellent performance on the TNO datasets, which further demonstrated its excellent generalization ability.

In conclusion, a large number of experiments on various datasets showed that our method can preserve a large amount of information from the source image and maintain the highlight degree of the infrared target in various complex situations. We attribute these advantages to the following aspects. On the one hand, the CMEF that we designed effectively extracts fine-grained information from source images and enhances the information interaction between different modalities. On the other hand, our network possesses a powerful feature extraction capability, and the ACmix module can effectively extract local and global information.

### 4.4. Ablation Study

We performed ablation experiments to validate the efficacy of various modules and utilized EN, Qabf, SF, MI, AG, and FMI_pixel for quantitative assessment. In addition, We selected two images for qualitative analysis, one of which was 00537D from MSRS and the other was 00390 from M3FD.

Quantitative analysis. We conducted quantitative experiments on the MSRS test set and summarized the results in Table 1. The values M1 and M2 represent changing ACmix to pure self-attention and convolution, respectively. The data in the table clearly show that the removal of the ACmix led to a decrease in all the indicators. The metrics, MI, SF, and FMI_pixel, showed the most significant declines, indicating a deterioration in the network’s ability to integrate complementary information between modalities. In addition, M3 and M4 denote the removal of HCGD in CMFE and the complete removal of CMFE, respectively. It can be seen that Qabf and AG experienced a large decrease when only HCGD was removed, which illustrated the effectiveness of HCGD in extracting high-frequency information. However, when the CMFE was removed, almost all the indicators significantly decreased, indicating that the performance of the network degraded a lot when there was no interaction between cross-modalities.

**Figure 9:**
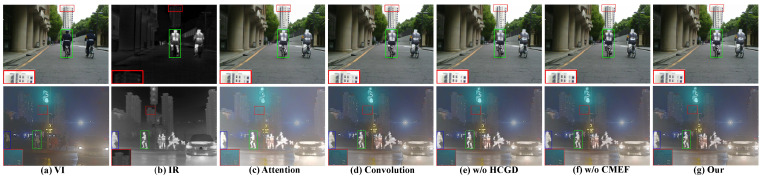
The results of ablation experiments.

Qualitative analysis. As observed in Figure 9, it is evident that the Attention was highly sensitive to whitening, leading to an overall increase in brightness and a loss of fine texture details. Since HCGD is one of the components of CMFE, removing both of them resulted in a significant loss of edges in the fused image. Moreover, removing the entire CMFE module had an even more significant impact, affecting not only the edges but also compromising the background and other critical information.

In summary, the results in Figure 9 and Table 1 indicate the effectiveness and rationality of our designed modules, as well as of the overall network design.

### 4.5. Downstream IVF Applications

In this section, we applied fused images to object detection and semantic segmentation, and explored the benefits of IVF for downstream tasks.

#### 4.5.1. Object Detection Performance

We employed the pre-trained YOLOv8 [52] detector to detect different images. We randomly selected a test set consisting of 160 images, with 80 images from the MSRS dataset and the remaining 80 images randomly selected from the M3FD dataset [12]. These 160 images contained a variety of scenes in the city, and we marked the most common objects among them, namely people and cars, as the objects to detect.

We assessed the detection performance of various methods using the mean average precision (mAP) metric. The results, indicating the mAP values at different IoU thresholds, are presented in Table 2. In addition, we calculated different mAPs, as in [53]. The prominent thermal target structure of the IR image helps the detector detect the human body, and the VI image can provide rich vehicle semantic information, so the detector can better realize the detection of vehicles. By fusing the two modalities of IR and VI images, performance in detecting both people and vehicles is enhanced. However, from the results, many algorithms tended to weaken the strength of salient objects, such as SwinFusion and SDNet, so their performance in detecting people was much lower than the recognition of source images. Taken together, our method was the best for person and car detection under almost all IoU thresholds.

**Figure 10:**
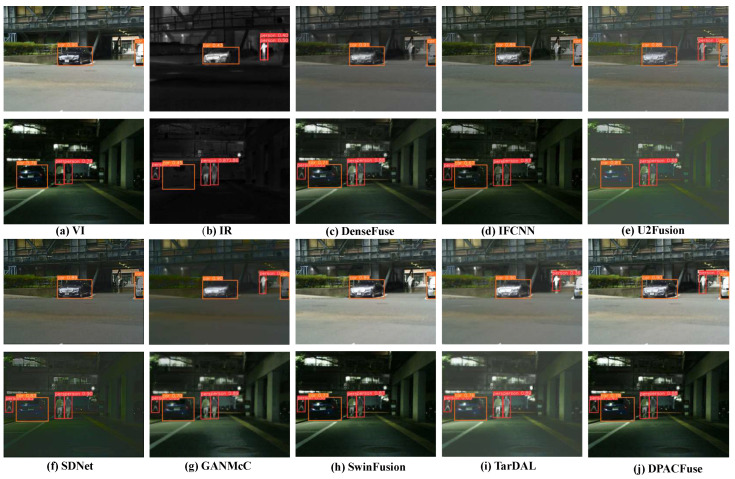
Object detection results on the MSRS dataset. The results are provided for two scenes from 00479D (**top**) and 01348N (**bottom**), respectively.

**Figure 11:**
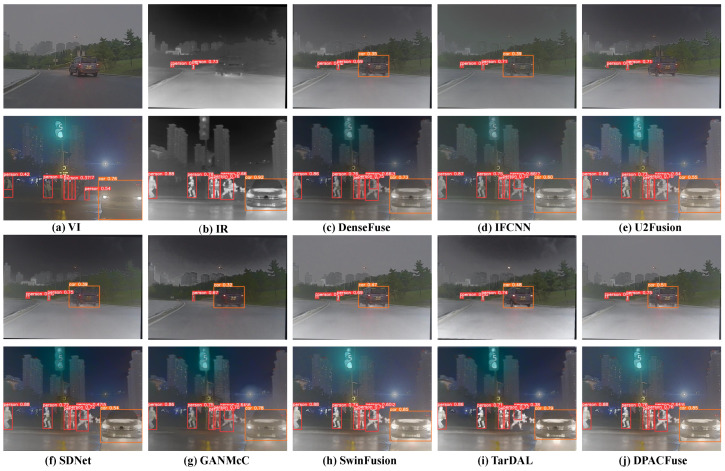
Object detection results on the M3FD dataset. The results are provided for two scenes from 01136 (**top**) and 00390 (**bottom**), respectively.

At the same time, we provide the detection results for visual display. As shown in Figure 10, in the scene in the 00479D image, due to insufficient illumination in the VI image, the DenseFuse, IFCNN, and SwinFusion methods could not detect the person in the image, and a similar situation also occurred in the scene in 01348N. In the scene in 01348N, although most of the methods successfully detected people and cars in the scene, the confidence levels were very different. In Figure 11, the situation is similar to that in Figure 10. Most methods could not identify people or cars due to the influence of illumination or distance factors. Only our method completely recognized people and cars in the scene and maintained a high level of confidence. This fully shows that the images generated by DPACFuse can provide rich semantic information for the object detector.

#### 4.5.2. Semantic Segmentation Performance

We performed semantic segmentation on the MSRS dataset. Specifically, we utilized source images and different fused images to train semantic segmentation networks [54], respectively. For more details on the semantic segmentation network, please refer to [21]. At the same time, we evaluated model effectiveness using Intersection-over-Union (IoU). The segmentation of each object is shown in Table 3. The results clearly demonstrate that DPACFuse achieved the highest performance in all categories of IoU. This outcome strongly indicates that DPACFuse effectively integrates information from IR and VI images, thereby improving the model’s ability in boundary perception, which leads to more accurate segmentation results.

**Figure 12:**
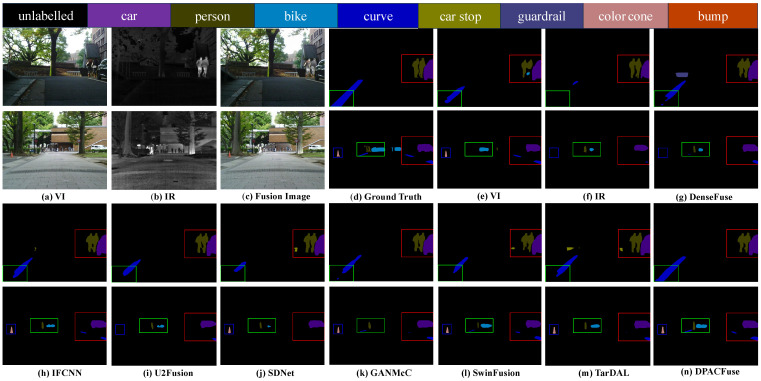
Visualization results of semantic segmentation on the MSRS dataset. The two scenes from top to bottom are from: 00055D and 00504N.

Furthermore, we also provide the segmentation results for visual presentation. As observed in Figure 12, the IR image exhibited good segmentation performance for persons, but it performed poorly in segmenting other objects, such as color cones and curves. In addition, insufficient illumination negatively affected the segmentation performance of the VI images. From the scenes in the two images in the figure, it is evident that our DPACFuse had an excellent effect on both the segmentation of people and the segmentation of other objects, which shows that the images generated by DPACFuse can better promote semantic segmentation.

## 5. Conclusions

In this paper, we propose a dual-branch progressive fusion framework, named DPACFuse, to be used for infrared and visible image fusion. Firstly, the Cross-Modality Feature Extraction we designed extracts inter-modality shared features as well as suppresses the loss of high-frequency information. Second, with the help of the ACmix module, our architecture more fully extracts the information in the source images for fusion. Finally, extensive experiments on three publicly available datasets showed that our DPACFuse outperforms all current state-of-the-art methods. In addition, in order to evaluate our approach more comprehensively, we also conducted experiments in two downstream tasks, object detection and semantic segmentation, and the results of the experiments further demonstrated the effectiveness and superiority of our approach.

## Figures and Tables

**Table 1:** Quantitative results of six indices under ablation experiments. In the evaluation results, the best-performing method is highlighted in red. The second-best result is represented in blue.

	M1	M2	M3	M4	Our
EN	6.6125	6.6146	6.6437	6.6017	6.6571
Qabf	0.6912	0.6820	0.6635	0.6507	0.7005
SF	11.2660	11.1202	10.9875	11.0457	11.3704
MI	4.5540	4.6525	4.4937	4.2439	4.7669
AG	3.6181	3.5809	3.3374	3.5419	3.7201
FMI_pixel	0.9289	0.9291	0.9277	0.9262	0.9314

**Table 2:** Object detection performance (mAP) of source images and the fused images of different fusion methods. In the evaluation results, the best performance method is highlighted in red. The second-best result is represented in blue.

	AP@0.5			AP@0.7			AP@0.9		
	Person	Car	All	Person	Car	All	Person	Car	All
IR	0.7891	0.5491	0.6691	0.7394	0.4787	0.6091	0.1961	0.1946	0.1953
VI	0.5478	0.7660	0.6569	0.3873	0.7108	0.5491	0.0359	0.3462	0.1911
DenseFuse [18]	0.7731	0.8079	0.7905	0.7296	0.7713	0.7505	0.1578	0.4583	0.3081
IFCNN [22]	0.7862	0.7768	0.7815	0.7316	0.7246	0.7281	0.1620	0.4118	0.2869
U2Fusion [31]	0.7823	0.7950	0.7937	0.7347	0.7724	0.7536	0.1599	0.4053	0.2826
SDNet [16]	0.7523	0.7649	0.7586	0.6519	0.7315	0.6917	0.1043	0.3826	0.2434
GANMcC [51]	0.7657	0.8132	0.7895	0.7250	0.7658	0.7454	0.1834	0.4415	0.3129
SwinFusion [13]	0.7699	0.7980	0.7840	0.6969	0.7499	0.7234	0.1183	0.3867	0.2525
TarDAL [12]	0.7897	0.7746	0.7822	0.7083	0.7246	0.7165	0.1646	0.4070	0.2858
DPACFuse	0.8034	0.8210	0.8122	0.7462	0.7753	0.7607	0.1862	0.4312	0.3087

**Table 3:** mIoU(%) values for segmentation semantics for different images on the MSRS dataset. In the evaluation results, the best performance method is highlighted in red. The second-best result is represented in blue.

Method	Background	Car	Person	Bike	Curve	Car Stop	Cuardrail	Color Tone	Bump	mIoU
IR	97.96	85.69	71.27	65.46	52.52	54.48	27.59	54.92	60.11	63.33
VI	97.69	83.07	54.67	66.27	51.97	54.94	59.69	50.35	66.18	64.98
DenseFuse [18]	98.36	89.48	73.47	69.84	57.76	63.56	65.07	62.34	66.00	71.76
IFCNN [22]	98.38	89.54	72.25	70.15	56.85	64.08	54.43	63.35	71.92	71.22
U2Fusion [31]	98.27	88.08	73.42	69.39	57.85	62.76	53.03	59.7	69.75	69.75
SDNet [16]	98.35	89.39	74.31	69.31	57.56	61.63	49.53	60.76	71.46	70.26
GANMcC [51]	98.34	88.85	73.68	69.67	56.75	65.17	57.06	61.50	71.72	71.41
SwinFusion [13]	98.25	88.08	70.74	68.66	74.31	61.70	67.34	64.06	67.86	71.67
TarDAL [12]	98.3	89.11	72.67	68.96	57.00	62.34	52.43	60.79	61.41	69.22
DPACFuse	98.6	90.38	74.58	71.94	65.39	74.44	84.66	66.03	77.18	78.13

## Data Availability

The authors confirm that the data supporting the findings of this study are available within the article.
